# Binder-free NiO/CuO hybrid structure via ULPING (Ultra-short Laser Pulse for In-situ Nanostructure Generation) technique for supercapacitor electrode

**DOI:** 10.1038/s41598-023-34274-w

**Published:** 2023-04-28

**Authors:** Mayuresh Khot, Rahaman Sharif Shaik, Wania Touseef, Amirkianoosh Kiani

**Affiliations:** 1 Silicon Hall: Micro/Nano Manufacturing Facility, Faculty of Engineering and Applied Science, Ontario Tech University, 2000 Simcoe St N, Oshawa, ON L1G 0C5 Canada; 2 Department of Mechanical and Manufacturing Engineering (MME), Ontario Tech University, 2000 Simcoe St N, Oshawa, ON L1G0C5 Canada; 3 Department of Manufacturing Engineering, School of Mechanical Engineering (SMEC), Vellore Institute of Technology (VIT), Vellore, Tamilnadu 632014 India

**Keywords:** Engineering, Materials science, Nanoscience and technology

## Abstract

Developing a cost-effective pseudocapacitor electrode manufacturing process incorporating binder-free, green synthesis methods and single-step fabrication is crucial in advancing supercapacitor research. This study aims to address this pressing issue and contribute to the ongoing efforts in the field by introducing ULPING (Ultra-short Laser Pulse for In-situ Nanostructure Generation) technique for effective design. Laser irradiation was conducted in ambient conditions to form a CuO/NiO hybrid structure providing a synergistic contribution to the electrical behavior of the electrode. Mainly, the effects of surface morphology and electrochemical surface because of tuning laser intensity were analyzed. The samples demonstrated high oxide formation, fiber generation, excellent porosity, and ease of ion accessibility. Owing to a less than 10-min binder-free fabrication method, the electrochemical performance of the as-fabricated electrode was 25.8 mC cm^−2^ at a current density of 1 mA cm^−2^ proved to be excellent. These excellent surface properties were possible by the simple working principle of pulsed laser irradiation in ambient conditions and smart tuning of the important laser parameters. The CuO/NiO electrode demonstrates excellent conductivity and rewarding cyclic stability of 83.33% after 8000 cycles. This study demonstrates the potential of the ULPING technique as a green and simple method for fabricating high-performance pseudocapacitor electrodes.

## Introduction

Renewable energy is a highly researched area in sustainability due to the need for zero-emission commitments. As global energy demand increases, finding ways to store this energy becomes crucial. One way to store energy is through electrochemical systems like batteries, capacitors, and supercapacitors. Electrochemical systems are widely used due to their portable and mobile energy storage capabilities. Electrochemical devices are differentiated by power density (dielectric capacitors) or energy density (rechargeable batteries). Supercapacitors occupy the space between batteries and capacitors; and are divided into Electrochemical Double-Layer Capacitors (EDLC) and pseudocapacitors. Research has shown that pseudocapacitors store one order greater charge, improving the energy density compared to EDLCs^1–7^.

Transition metal as an active ingredient remains popular in pseudocapacitors research. Transition metals in various states such as oxides, chalcogenides, chalcogenides, diselenides, MOFs, COFs, hydroxides, and MXenes are widely used in research for high-energy–density pseudocapacitors electrodes^8–12^. As a pioneer transition metal, RuO_2_ was proven to be a promising material due to its performance matching near theoretical capacitance; however, the high cost, non-abundance, and toxicity made the material non-ideal^13^. This allowed for further research for other attractive transition metals such as MnO_2_, TiO, Co_3_O_4_, ZnO, NiO, and CuO, which are commonly being investigated to find a cost-effective, abundant, and electronically conductive electrode active ingredient for fabrication^14–16^. The capacitance of pseudocapacitors can be increased by increasing the surface area of the fabricated metal-oxide electrode. This is because most energy storage depends on the surface redox reactions as well as charge accumulation due to EDL at the interface^17^. The enhanced surface area will permit improved active sites for redox, allowing for improved rate capability and maximum ion adsorption at the surface for improved charge storage^18–22^. Some recent pseudocapacitor research involved CuSe–TiO_2_–GO ternary nanocomposite which was proposed by Sajjad et al. as a solution to the challenge of simultaneously achieving high capacitance and widened voltage frames in an aqueous supercapacitor system. The ternary nanocomposite was tested and found to have a remarkable capacitive response, high specific energy, superior specific power, and excellent stability, making it a promising material for hybrid supercapacitors^23^. Another study by Sajjad et al. presents the development of novel CdO–rGO nanocomposites as positive electrodes for hybrid supercapacitor devices (HSs) with large energy storage capacity and low operating costs. The optimal composite with 25% rGO concentration demonstrated excellent specific capacitance and rate performance, leading to the development of a high-performing CdO-25%rGO//AC HSC with high specific capacitance, large energy density, and excellent stability^24^. Another spectrum of the research has also focused on interesting research such as self-charging supercapacitor power cells by piezo-ionic effect by Pazhamalai et al. in this work, the MoS_2_-Nafion-MoS_2_ SCSPC has shown to possess high capacity/capacitance, energy density, and long cycle life, and the self-charging characteristics of the MoS_2_–Nafion–MoS_2_ SCSPC^25^. Similarly, the work of Mishra et al. a CuO–NS_2_-based supercapacitor showed 60% charged potential retention and was used in a demonstration of wireless power transmission in burst mode, showing its potential for use in power management systems^26^.

All the recent progress redirects to newer chapters of supercapacitor technology. The research also demands fast fabrication of supercapacitor electrodes^27^. In this study we investigate the hybrid structure of NiO/CuO and its charge storage abilities for its use in supercapacitors. Ni and Cu are widely used in electrical applications due to various beneficial characteristics such as high theoretical capacitance, excellent electrical conductivity, earth-abundant, nontoxicity, and affordability, making them a desirable choice for material selection^28,29^. Various research has already been conducted on forming hybrid CuO/NiO structures. Such as the research carried out by Zhenbin et al. detailed the utilization of NiO–CuO mesoporous nanowire array on Ni foam substrate using the hydrothermal method and post-annealing process. The fabrication technique employed in the method was expensive and required post-annealing, making the process/technique time-consuming^30^. Chakraborty et al. prepared a mixture by hydrothermal synthesis. Researchers reported the composite structure of CuO/NiO showed up to 83% capacitance retention after 1500 charge–discharge cycles, which remains satisfactory. But these techniques involve a binder of three to four structures, which often makes the method complex and expensive^31^. Huang et al. utilized composite structures by transferring them to a Teflon-line autoclave and prepared NiO nanoflake-coated CuO flower core–shell nanostructures^32^. Although the work proved promising results, the synthesis involved waste such as ammonia derivative which may find difficulties in large-scale production.

Tremendous research is still being continued to find the best performing capacitive electrode for pseudocapacitors; however, significant challenges and shortcomings remain in the fabrication method currently being utilized, which are either time-consuming, requiring post-processing treatment, binder adhesion of active material, harmful precursors and the tremendous waste being generated which does not seem feasible for scalable and mass production. Finding the best manufacturing method focusing on green synthesis remains the top priority^27,33^. Applying a green nano synthesis technique like pulsed laser irradiation into the fabrication process can solve the quest to find a binder-free fabrication technique that can deliver increased surface area, enhanced porosity, and distributed surface morphology for pseudocapacitance electrodes^34–38^. The architecture of the surface is governed by the simple working principle of laser. This uniqueness in the ULPING technique lies in its fabrication process in which the phase conversion of the irradiated sample occurs in ambient conditions. Unlike other synthesis methods such as pulsed laser deposition and physical/chemical vapor deposition, this technique does not require a controlled environment or gas inflow^39^. Although the ULPING technique may share common grounds with laser ablation, we do not focus on the removal of material but rather on an in-situ phase conversion of substrate material into an oxidized surface. Therefore, this technique is an in-situ and self-deposit technique. The main challenge with the ULPING technique is the evaporation of fiber and the formation of large granules which can happen when the photonic energy from the laser surpasses the mechanical properties of the irradiated material^40–42^. All parameters in the fiber laser which is being used for this study as seen in Table 1 are significant, therefore, a series of trials and errors can be conducted to obtain the optimal range for the treated material. Given the simple working principle of oxide formation on transition metals with pulsed laser irradiation, this fabrication process can be revolutionary as the mass scalable production process.

**Table 1:** Laser parameters for different samples.

Sample	Scan speed (mm/s)	Pulse repition Rate (KHz)	Power (W)	Pulse width (ps)	Pulse energy (μJ)	Peak pulse power (kW)	Fluence (J/cm^2^)	Laser intensity (kW/cm^2^)
P8	10	1200	**8**	150	6.667	44.444	0.133	888,889
P12	10	1200	**12**	150	10.000	66.667	0.200	1,333,333
P15	10	1200	**15**	150	12.500	83.333	0.250	1,666,667
P20	10	1200	**20**	150	16.667	111.111	0.333	2,222,222

Significant values are in [bold].

## Experimental setup and methodology

### Material synthesis and preparation of electrodes

**Figure 1:**
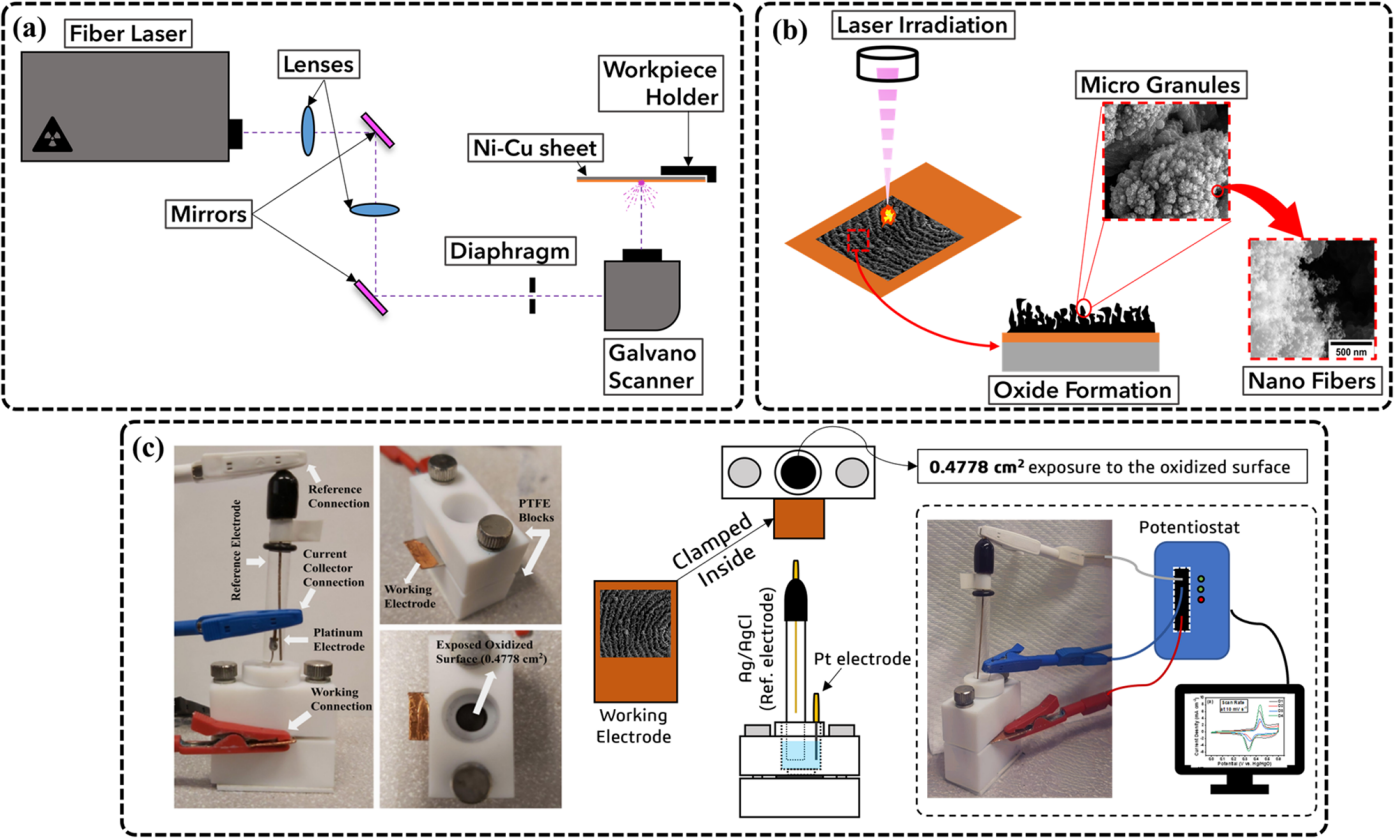
(**a**) Complete setup of the fabrication (**b**) Laser irradiation via ULPING technique generates nano morphology ideal for charge storage (**c**) Three-electrode setup.

A 0.3 mm Ni sheet was acquired from Sigma Aldrich. The Ni sheet was cleaned with DI water and acetone to eliminate any residue from manufacturing and transport. Carefully handled, a commercially available high-purity conductive Cu tape was applied onto the Ni sheet, ensuring no air pockets and creases were visible. The tape-covered sheet was wiped with a Kimwipe to remove any smudges and debris. The prepared Cu-taped Ni sheet was then situated on an XYZ free-motion plate. A circular profile was designed in MarkingMate (CAD software), and the laser parameters were set on the laser software, as shown in Table 1. The setup of the laser and all the essential components such as mirrors and optical glass are best demonstrated in Fig. 1a. Upon satisfactory focal length from the preview profile, the laser irradiation was followed. A better visual is provided in Fig. 1b of the complete process. The wavelength of the picosecond was 1064 nm. The as-obtained samples were cut into smaller, rectangular pieces with the irradiated circle on one end. Similar steps were carried out for coin cell setup as previously mentioned; however, two circular coin cells of the same laser parameters were punch holed from the Cu-taped Ni sheet for symmetric electrochemical testing. The nomenclature of the samples was assigned as shown in Table 1.

### Material characterization

As for the nanoscale analysis of the morphology and structural characteristics, SEM was conducted at a magnification of × 500, × 10,000, × 35,000, and × 100,000. The surface's micro and macro-scale analysis could be possible from the SEM images. To further analyze the microscopy results in nano regime, TEM was conducted. To understand the material properties and elemental composition of the created samples, EDX was conducted. In the EDX data, the presence of oxygen would play an important role as the surface desired is to be highly oxidized. In addition to material composition, XRD was conducted to analyze the diffraction pattern, crystalline structure of the hybrid samples. Lastly, XPS was also conducted to understand the ratio of oxidation states that were present after the sample preparation.

### Measurement of electrochemical properties

A three-electrode setup was utilized as shown in Fig. 1c. For the three-electrode configuration, the use of a Plate Material Evaluating Cell (PMEC) obtained from ALS Co., Ltd and was utilized. Two PTFE blocks sandwich the working electrode, with the top block exposing the laser-irradiated, oxidized part only. Part of the electrode sheet was exposed outside of the sandwiched block for connection to working; an Electrolyte of 0.5 M KOH was used as an ionic solution. Ag/AgCl reference electrode was acquired from ALS Co., Ltd, and an auxiliary platinum wire electrode obtained from DEK Research was used as a current collector. An SP-150 potentiostat was used to conduct electrochemical tests such as Cyclic Voltammetry (CV), Galvanostatic charge–discharge (GCD), and Electrochemical Impedance Spectroscopy (EIS) on the prepared samples. For the two-electrode setup, an MTI split test cell was used in which the coin cells were placed with a simple 2 M KOH electrolyte-soaked separator sandwiched between the two-coin cells. Simple tests such as CV, GCD, and cycle retention were conducted to examine the electrochemical behavior of the symmetric cell.

The calculation of specific capacity was done using Eq. (1) for three-electrode setup. Here, *J* is the current density which can be calculated by dividing area, *A* from current, *i*, and $\Delta t$ is the discharge time.

1 $$ Q = J \times \Delta t, \;where\, J = \frac{i}{A} $$

## Results and discussion

### Morphology and structural properties

**Figure 2:**
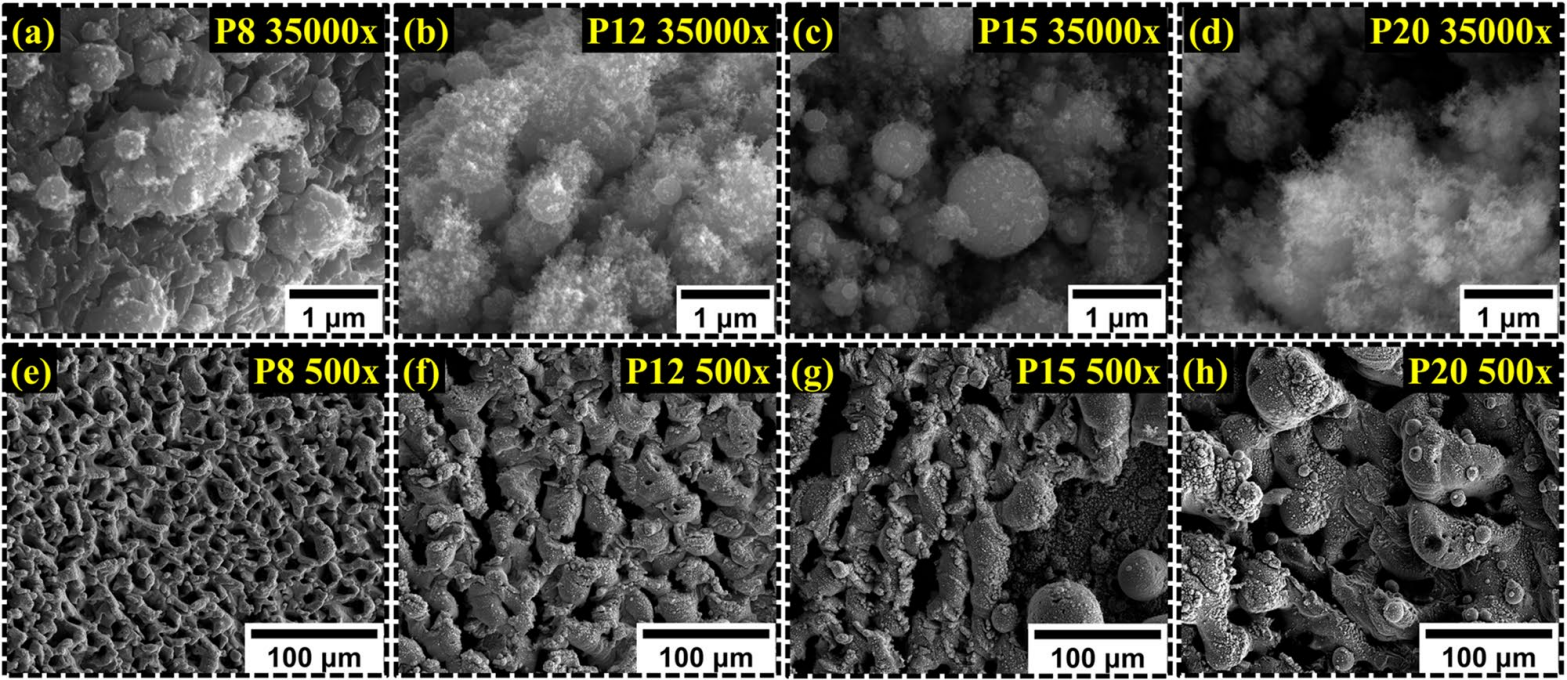
(**a**–**d**) The higher magnification demonstrates nanostructure growth such as nanofibers. (**e**–**h**) The lower magnification reveals critical features such as surface distribution, accessibility, granule size, and voids.

Upon visual inspection of the laser-treated samples, a relationship was observed. As the average power was increased, oxidation and mass ablation were evident. For P8, the Cu on the Cu tape was oxidized, demonstrating a fragile film of CuO present on the substrate. For P12, the Cu tape was largely oxidized and converted into a thin film with a bit of oxidized Ni which was visible. Going into the higher intensity, visually, dark oxidized color with a hint of copper was noticed, indicating the presence of both NiO and CuO. SEM was conducted under the 4 samples to find the topology, porosity, surface area, and presence of nanostructures. Starting with P8, the higher magnification shown in Fig. 2a demonstrated microstructure with granules attached to the surface and no presence of nanostructures. In higher magnification, however, the surface was more uniformly distributed and showed excellent porosity as demonstrated in Fig. 2e. The lower intensity of the laser beam allowed less pulse energy to be transferred to the substrate promoting rapid quenching due to the presence of a large temperature gradient in ambient conditions. Progressing to P12, P15, and P20, as seen in Fig. 2b–d, the nanofibrous growth was more evident. P12 and P20 demonstrated the most nanoscale fiber growth of the hybrid electrode, with P12 demonstrating fibrous growth throughout. The lower magnification of the microscopic image shows more considerable granules growth as the power is increased. This is due to the agglomeration effects of reaching near plasma temperature^43,44^, as seen in Fig. 2f–h. As the macroporous structural properties also dictate the better rate capability of a pseudocapacitors cell, P12 demonstrated far better uniformity in surface distribution, porosity, and better surface area compared to P15 and P20. For a rough estimation of the porosity levels, ImageJ was utilized by adjusting the threshold of the SEM images. The porosity levels for P12, P15, and P12 were 40%, 36% and 21% respectively. Overall, P12 demonstrates all the required surface properties ideal for design^45^. We determined the specific surface area of the fiber through ImageJ analysis, as presented in Table 2. The fiber dimensions were obtained from TEM images, assuming the fibers to be cylindrical in shape^37^. Although there may be some margin of error due to these assumptions, the values in Table 2 provide a clear indication of the trend in specific surface area, which aligns with changes observed in both material and electrochemical properties.

**Table 2:** Specific Surface Area of fiber on samples.

Sample	Specific surface area (m ^−1^)
P8	0.019558
P12	0.099484
P15	0.003516
P20	0.066065

**Figure 3:**
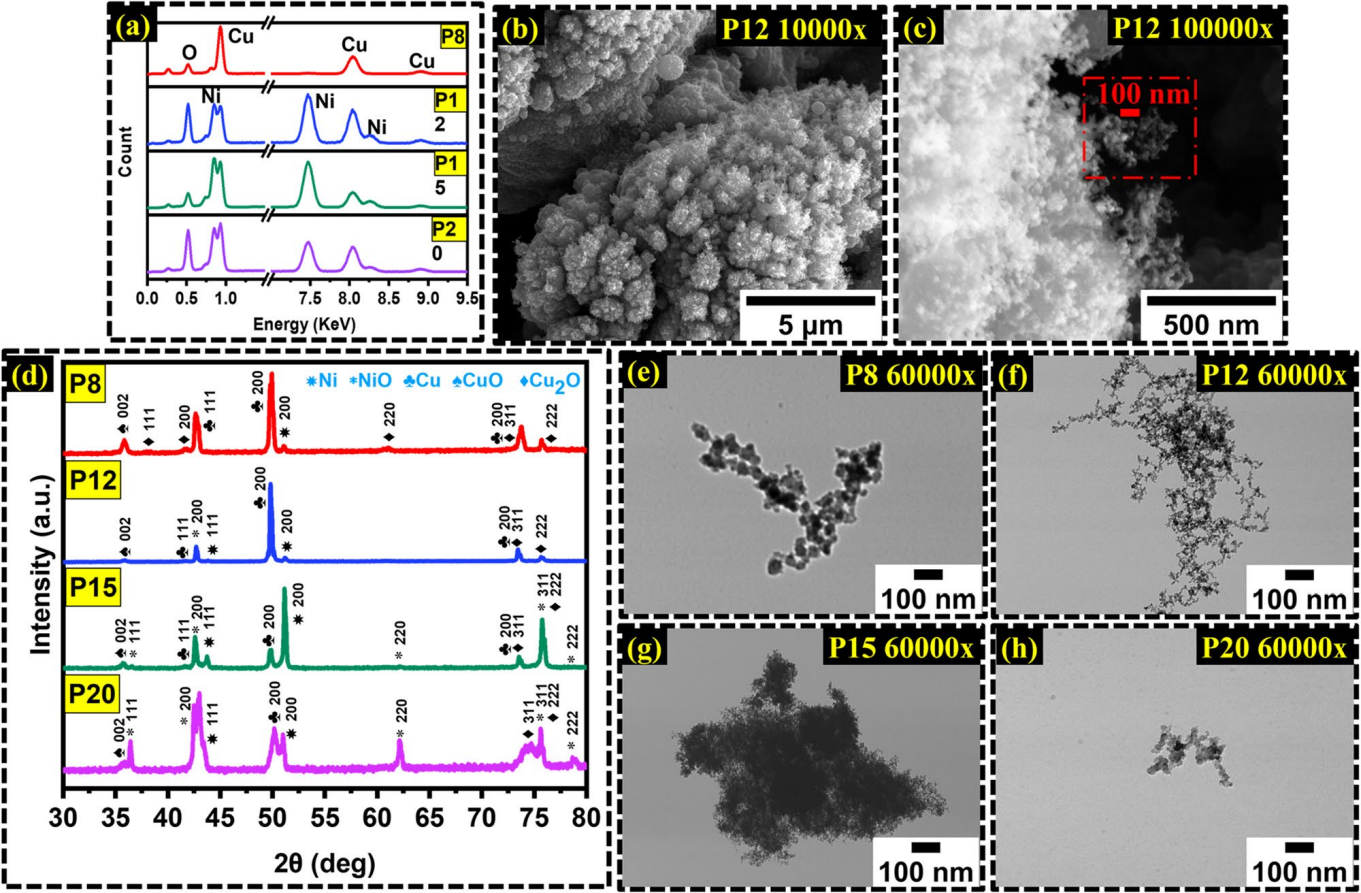
(**a**) The EDX data demonstrates elemental composition and the presence of oxygen caused during laser irradiation. (**b**) The SEM image of P12 with × 10,000 magnification shows fibrous growth on the granule structure. (**c**) An × 100,000 magnification approves of nanoscale fiber growth (**d**) XRD diffraction pattern for all samples (**e**–**h**) TEM sample of P8, P12, P15 and P20.

EDX was conducted on the basis to discover the elemental composition to prove the presence of oxygen due to the oxidation process via laser irradiation as shown in Fig. 3a. P8 shows low oxygen detection and high copper presence with no traces of Ni. However, the 12W power sample, P12, demonstrated a high oxygen presence along with Ni and Cu, indicating the formation of a hybrid structure from the irradiation. The high-power samples, P15 and P20, showed similar results to P12; however, the presence of oxygen wasn’t as dominant as in P12. A closer look at the P12 sample with SEM shows that the morphology inhabits granular and fiber growth on the surface through × 10,000 magnification. The fiber is grown in a granular shape and exhibits an urchin-like morphology shown in Fig. 3b. This morphology follows the microporous structure requirements essential for enhanced surface area for maximum charge storage and provision for maximum redox-active sites. At × 100,000 magnification demonstrated in Fig. 3c, the morphology confirms the presence of nanoscale growth of the active material, proving the ULPING technique is successful and efficient for the electrode fabrication method.

X-ray diffraction pattern were analyzed for analysis of crystalline properties and the overall structure of the samples. With the increase in power, the collective contribution of Ni and Cu are observed in the hybrid structure from the diffraction peaks. The two common phases of copper oxide, Cuprous oxide, Cu_2_O and Cupric Oxide (CuO) were discovered. In addition, diffraction patterns of plain Cu were also highlighted in the survey. Both crystal system of cubic and monoclinic were observed from both copper oxide phases. Figure 3d highlights the index reference to each corresponding angle as referred to the JCPDS card No for Cu_2_O and CuO (05-0667 and 45-0937 respectively). As the laser power is increased, the inclusion of cubic NiO phase becomes more apparent. This aligns with the XRD pattern of standard cubic NiO crystalline structure^46–50^. TEM was conducted on all samples; the results of TEM closely conform to the findings in SEM. The improvement in surface area can be assessed by the fiber diameter. P8 shows fiber strand diameter of 70 nm obtained by taking the average as depicted in Fig. 3e. P12 on the other hand showed remarkable interlinked fiber with strand diameter being roughly 15 to 20 nm in size as can be noticed in Fig. 3f. This proves 12 watts being the optimal power setting for maximum fiber growth for hybrid structure. Figure 3g of P15 sample shows no significant fiber interlinked, however, maximum oxidation can be assumed due to particle agglomeration. Additionally, SEM images showed minimal fiber growth in P15 which agrees with TEM findings. Lastly, P20 showed the next best nano regime particle or fiber size. By averaging the fiber diameter, 25 nm is ideal assumption for P20 as shown in Fig. 3h.

**Figure 4:**
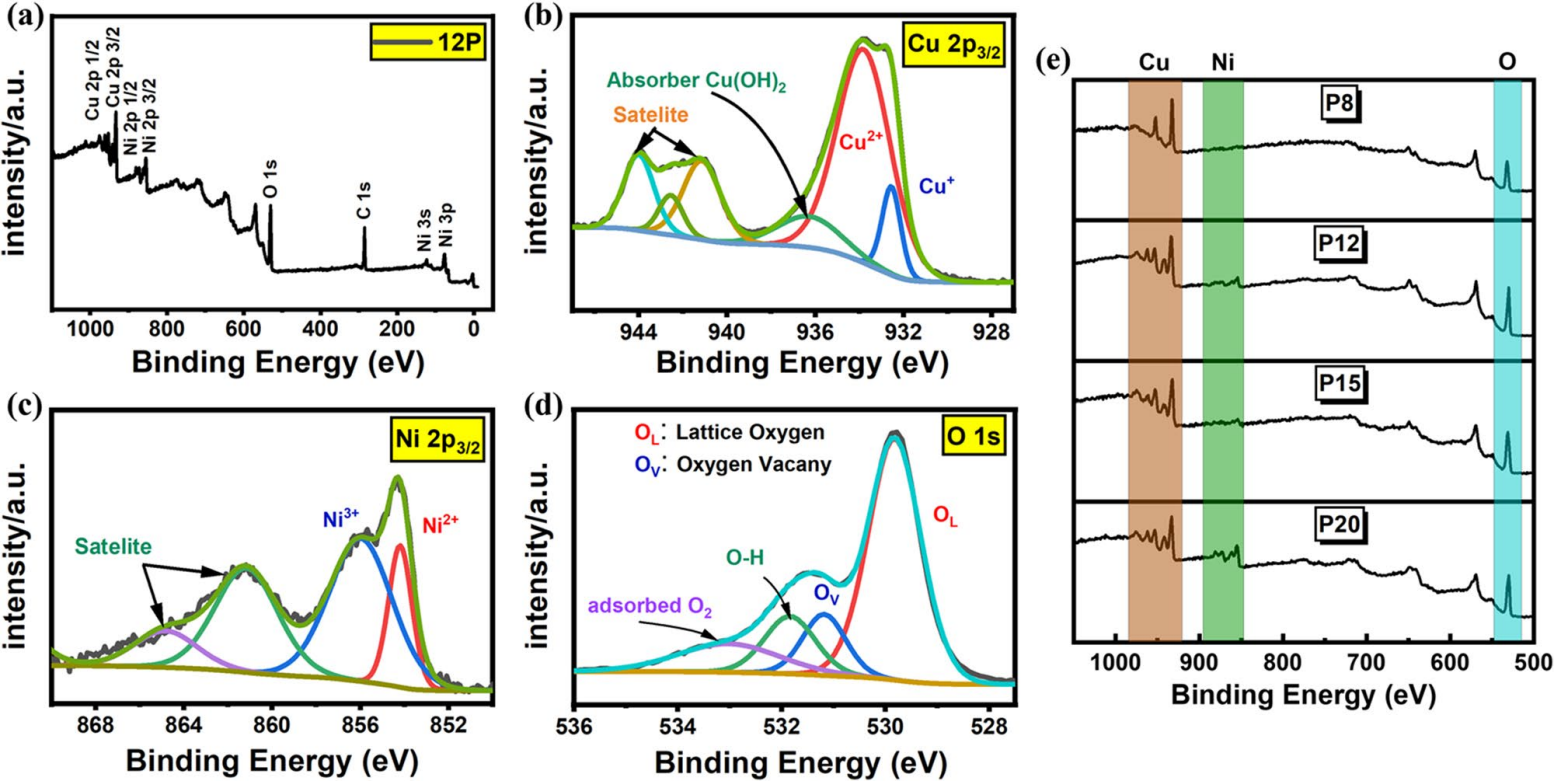
(**a**) XPS survey to define material composition (**b**) High-resolution XPS for Cu 2p_3/2_ (**c**) High resolution of XPS of Ni 2p_3/2_ (**d**) O 1 s XPS signal deconvolution (**e**) Progression of Ni formation with increased power.

XPS was conducted on all four samples. Figure 4a illustrates a survey of all the species present on the surface layer via a complete XPS spectrum. The peaks of Cu 2p_3/2_, Ni 2p_3/2,_ and O 1 s were dominant and each was deconvoluted into many signals at higher resolution spectra to find surface oxidation. The XPS spectra of core level Cu 2p present satellite structure in the higher energy range indicating the presence of the CuO phase. This satellite shake-up structure was present in all samples except for P8. The Cu 2p_3/2_ was deconvoluted into three signals as shown in Fig. 4b of which the ~ 932.7 eV was ascribed to Cu^+^, ~ 933.6 was ascribed to Cu^2+^ and the third peak was ascribed to ~ 935.6 denoting copper hydroxide (Cu(OH)_2_) species on the surface layer. From the XPS data of Cu 2p_3/2_ core levels, as the energy density of the laser to carry out irradiation increased, the Cu^2+^ phase was less dominant and the Cu^+^ phase became more apparent on the surface layer. Observing the Ni XPS high-resolution spectrum presented in Fig. 4c, The Ni 2p_3/2_ peak was analyzed to find the Ni oxidation states. Like Cu 2p_3/2_, Ni 2p_3/2_ demonstrated a shakeup satellite structure at higher energy levels. The Ni 2p_3/2_ peaks were deconvoluted into two signals of which the peak at ~ 854.2 eV was ascribed to the Ni^2+^ phase of NiO and the second signal at ~ 855.6 eV was ascribed to the Ni^3+^ state of Ni_2_O_3_. Going from P12 to P20, it was observed that increasing energy density allowed for an increase in the Ni^3+^ phase compared to the ratio of the Ni^2+^ phase. Finally, the XPS peak of O 1 s core level was analyzed. Here, the peaks were deconvoluted into four sub-peaks. The signals were identified as shown in Fig. 4d. The intense signal of peak 1 is ascribed to lattice oxygen, O_L_ at ~ 529.6 eV responsible for the bonding of O–Ni or O–Cu. Peak 2 at ~ 531.2 eV is ascribed to oxygen vacancy or mental deficiency. Peak 3 at ~ 531.8 eV ascribes to O–H group bonding and finally, peak 4 at ~ 533 represents the adsorbed O_2_ elements. In the case of all the samples, the lattice oxygen, O_L_ bonding environment is the most dominant compared to oxygen vacancies, O_V_. On all, the data demonstrated in the XPS conforms with the findings of EDX and SEM. T proves the effects of pulse energy, fluence, and laser intensity, a survey of all four samples are presented in Fig. 4e in which as the energy intensity of the laser beam increases, the peak of Ni becomes more apparent. Great energy intensity allows for deeper penetration of the sample with CuO being successfully fused onto the NiO structure and abundant ionization with surrounding O_2_ to form a hybrid structure. P8 demonstrated no traces of Ni since the lower energy intensity of the beam was equipped which was not able to penetrate through the Cu layer and ablate the Ni surface^51–59^.

## Electrochemical properties

Electrochemical tests were conducted to confirm the finding of structural characterization results, and observations were made. A series of tests such as CV, GCD, and EIS was conducted to analyze the electrochemical behavior of the laser-fabricated electrode via our ULPING technique.

**Figure 5:**
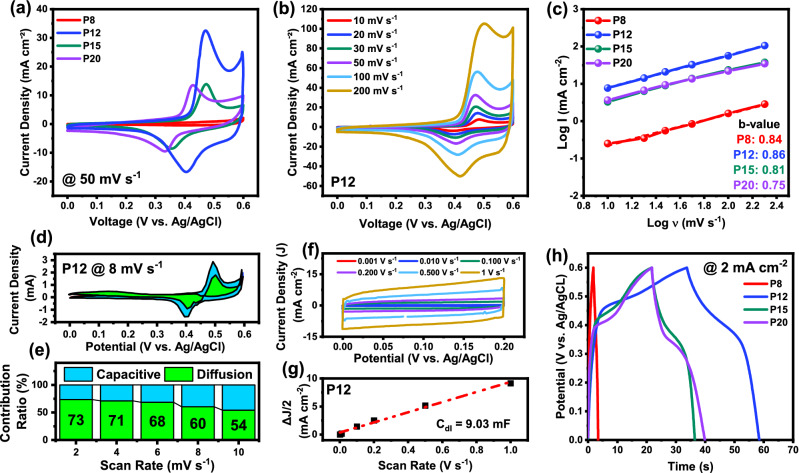
(**a**) CV profile of all four samples presented at 50 mV s^−1^ (**b**) CV profile of P12 under multiple scan rates (**c**) log (**i**) vs log (scan rate) to find b-value using power law (**d**) A separation of diffusion controlled and surface capacitive contribution of P12 CV (**e**) Bar chart defining the contribution ratio of P12 at increasing scan rates (**f**) CV profile of NiO/CuO hybrid structure of P12 in capacitive narrow voltage window (**g**) The ECSA calculated by taking current density in f and plotting against scan rate (**h**) GCD of all samples with P12.

CV was conducted on the sample to understand the charge storage mechanism of the fabricated electrode. A series of scan rates were performed on all the samples, of which 50 mV s^−1^ is presented in Fig. 5a. The stable, chosen potential for CV testing was 0 to 0.6 V in 0.5 M KOH, where no gas evolution reactions were visible. Unique redox reactions were observed as the power samples varied. P8 sample to which thin CuO film was generated due to the lowest pulse energy presented a non-uniform CV curve to which the redox peaks were not visible. However, due to the non-rectangular CV shape, pseudocapacitance was confirmed. The 8W power sample demonstrated the least charge storage and minimal rate capability due to the absence of nanoscale structures on the surface, unlike the high-power samples. The unique CV of the lower power samples conforms with the findings of EDX, in which the detection of Cu was significant; however, Ni was least observed. This is due to the pulse energy of the laser being relatively low, therefore not allowing the pulses to penetrate through the CuO film. The redox peak, however, was sharply dominant beyond the P8 sample. P12 demonstrated the maximum charge storage among all samples due to the optimal pulse energy for pulses to penetrate through the CuO. The Cu tape on the 12W sample was oxidized and held onto the surface of the Ni substrate, and the Ni was also oxidized under the Cu tape. As a result, the dominant curve from CuO and NiO redox is visible. A complete composite fusion process is visible in P15 and P20 samples in which the CuO nanoparticles are deposited onto the NiO. P12, however, demonstrated to exhibit the best performance. A series of scan rates were conducted on P12, as shown in Fig. 5b. An increase in the capacitive behavior was visible as the scan rate increased. Since the samples portrayed nanoscale structural formation and enhanced large surface area, the combinational effect of charge storage due to faradaic reactions and non-faradaic, surface charge accumulation defines the pseudo-capacitor equal working mechanism. To understand the charge storage mechanism of the fabricated samples, Dunn’s method was employed as denoted by the power law equation presented in Eqs. (2) and (3)^60,61^.

2 $$ i = ab^{v} $$

3 $$ \log i = \log a + b\log v $$

Here, *i* is the peak current from anodic curves, *a* and *b* are adjustable variables and *v* is the scan rate. The b-value is calculated from the slope of log (i) vs log (scan rate) for multiple data sets of peak current at various scan rates. A b-value close to 0.5 governs a diffusion-controlled working mechanism whereas a b-value closer to 1 exhibits capacitive or surface-controlled charge storage. From this analysis, as shown in Fig. 5c, the b-value of P12 was 0.86 which is ideal for pseudo-capacitance. To further quantify the contribution of the charge storage mechanism a series of CVs at slow scan rates were conducted from which Eq. (4) can be applied to separate the capacitive and diffusion behavior in the CV profile. Here the summation of *k*_1_*v* and *k*_2_*v*^1/2^ defines the capacitive and diffusion-controlled behavior to make up the current as a function of voltage.

4 $$ I\left( V \right) = k_{1} v + k_{2} v^{1/2} $$

Rearranging Eq. (4) yields,

5 $$ \frac{I\left( V \right)}{{v^{1/2} }} = k_{1} v^{1/2} + k_{2} $$

By plotting *I*(*V*)/*v*^1/2^ vs. *v*^1/2^, a best-fit line connects the data points from which values of *k*_1_ and *k*_2_ are representative of slope and y-intercept respectively. Carrying this process at various potentials provides a separation profile for both capacitive and diffusion processes. In Fig. 5d, the diffusion process is separated from the CV curve of P12 at 2 mV s^−1^. Once known of *k*_1_ and *k*_2_ value areas are, an *i*_d_ can be easily obtained by multiplying the desired scan rate. In Fig. 5e, as the scan rate increases, the capacitive nature becomes more apparent^62,63^.

For calculating the ECSA or the electrochemical surface area of P12, a capacitive dominant narrow window was analyzed from the previous CV curve to be 0.0 V to 0.2 V. Multiple CV scan rates from 0.001 V s^−1^ to 1 V s^−1^ were conducted as shown in Fig. 5f. The resultant change between anodic and cathodic current density was found for each scan rate and the graph was plotted as demonstrated in Fig. 5g. A line of best fit provides the slope which represents the double-layer capacitance or C_dl_ which was 9.03 mF for P12. Comparing this with the second-best performer in this work P20, the C_dl_ for P20 was only 2.71 mF. Assuming the C_sp_ to be 0.040 mF cm^−2^ which is typical for NiO, the ECSA of P12 is 225.75 cm^2^ as compared to 67.75 cm^2^ of P20^64–66^.

GCD tests of all samples were conducted at 2 mA cm^−2^ as presented in Fig. 5h. P12 demonstrates superior performance as compared with other samples. This was once again possible due to many reasons given above such as excellent surface area, porosity, and double-layer interfacial charge storage all contributing to the excellent performance of P12.

With all the tests, it was obvious to understand that the laser intensity and fluence have a direct relationship with each other as well as a close relationship with the morphology in creating a hybrid structure. Too much pulse energy assures excellent formation of hybrid structure, but at a loss of surface area. These effects mainly occur due to the aftereffects of pulse bombardment at the local spot. Due to high thermal diffusivity because of dense pulse intensity, the melt pool temperatures exceed the spot size which promotes granule agglomerates as well as the evaporation of nanofiber^67^.

**Figure 6:**
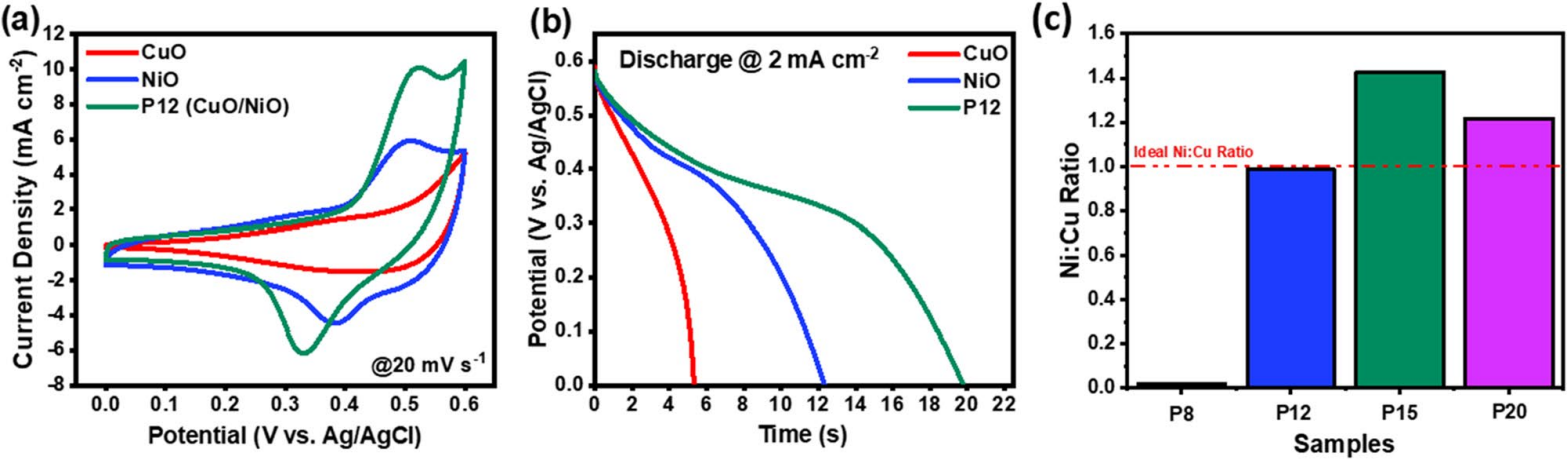
Analysis of synergistic effects by carrying individual analysis with same laser parameters as the best sample P12 (**a**) CV profile (**b**) GCD (**c**) Ni:Cu ratio identified through EDX.

To prove the synergistic effects taking effect in the P12 sample, a copper and nickel sheet were irradiated with the same parameter settings used for P12 using the ULPING technique (Refer to Table 1). The thickness of the sheet and the supplier of the sample were all the same and claimed 99% purity of material. For these samples, CV and GCD were run on the samples. The results are demonstrated in Fig. 6a and b. Figure 6a shows CuO with minimal redox peaks, however having a horn-like structure at 0.5–0.6 V. Similarly, the nickel oxide CV profile demonstrates faradaic behaviour with prominent redox peaks. The CV profile of P12 demonstrates collective contribution in which the horn-like profile is seen at 0.5–0.6 V, similar to CuO and the reversible redox peaks from NiO. GCD was also conducted on the same samples. The P12 samples has a large depletion time of charge as seen in Fig. 6b. This proves that the hybrid structure provides collective contribution in proving better performance.

P12 exhibits superior performance compared to its constituents (CuO and NiO) and other samples due to a variety of factors. In comparison to the individual metal oxides, the Cu^2+^ and Ni^2+^ ions present in the CuO-NiO nanoparticles possess partially-filled 3d-orbitals, which facilitate more efficient engagement in redox processes. This enhanced redox activity contributes to the superior performance of P12. According to various studies reported in the literature, an optimal Ni to Cu ratio of 1:1 is critical for achieving optimal electrochemical performance^30,68^. P12 exhibits superior performance due to the optimal Ni:Cu ratio obtained through 12W laser irradiation, as evidenced by the EDX data presented in Table 3 and Fig. 6c. The ratio of Ni:Cu is determined by the fluence and pulse intensity delivered to the substrate via the laser, as well as the material characteristics that are altered in response to the supplied heat. Furthermore, as confirmed by TEM and SEM analyses, P12 exhibits an enhanced surface area with an excellent nano fiber network, resulting in increased exposure to active surface redox sites at the interface^32,69,70^. This characteristic further contributes to the superior performance of P12.

**Table 3:** Ni:Cu ratio acquired from EDX.

Sample	Ni	Cu	Ratio
P8	1.38	67.66	0.0203
P12	29.53	29.97	0.9852
P15	26.93	18.86	1.4279
P20	38.3	31.45	1.2178

**Figure 7:**
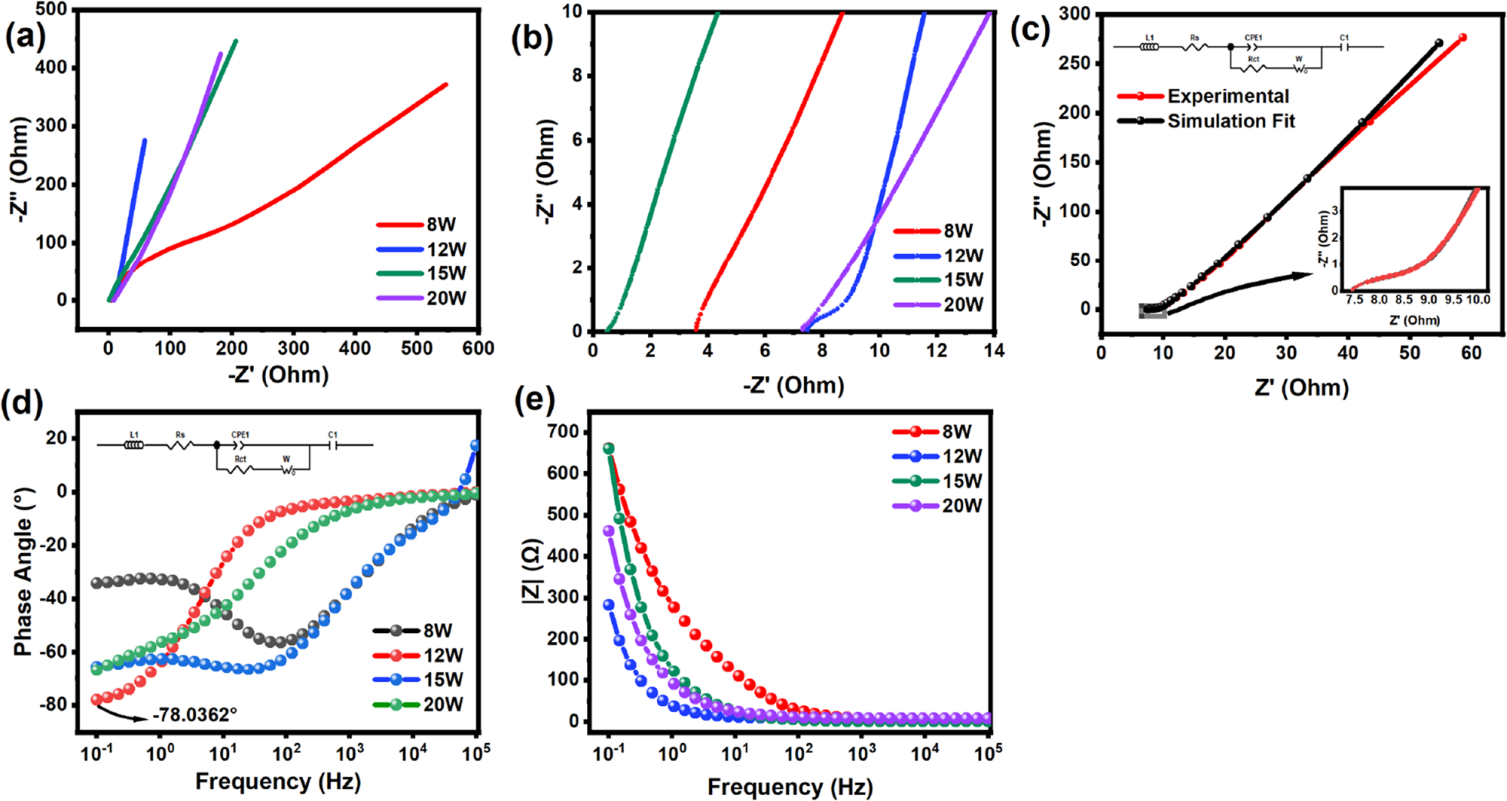
EIS was conducted on all samples to understand electrode conductivity (**a**) Nyquist plot shows P12 with near 90-degree slope at a lower frequency (**b**) High-resolution EIS for all samples to understand internal resistance (**c**) Simulated fit of P12 sample (**d**) Bode plot indicate P12 reaching near 90 degrees (**e**) Absolute impedance vs frequency curve proving least electrode resistance for P12.

EIS was conducted to find the electrochemical behavior of the samples in AC analysis. The frequency range was picked from 100 MHz to 100 mHz, and an equivalent circuit was designed that best relates to the fabricated setup. The equivalent open circuit is a traditional Randles circuit with a series connection of constant phase elements followed at the end. The Nyquist plot is shown in Fig. 7a. The higher frequencies demonstrate all the electrochemical resistance, such as series and charge transfer resistance, followed by the diffusion of ions into the electrode presented in the mid-frequency range. At lower frequencies, interfacial charge storage is analyzed. P8 had high internal resistance of both series and charge transfer presented by the depressed semi-circle and ion-diffusion slope at mid-frequency. Past the semi-circle, P8 showed a semi-infinite medium diffusion or the Warburg impedance with a 45°-line exhibiting battery-like behavior. The high impedance or poor conductivity is due to the inferior surface area required for better rate capability of the pseudocapacitor electrode and much-needed EDL formation for capacitive contribution.

On the other hand, the higher power samples such as P12, P15, and P20 were relatively closest to the finite space, demonstrating solid capacitive characteristics. P12, amongst all the other samples in lower frequency, demonstrates excellent capacitive behavior due to its near 90°. The line at lower frequencies. From Fig. 7b a closer look at the internal and diffuse layer resistance can be analyzed; P15 exhibits the lowest internal resistance. This is due to the successful laser pulse ionization fusion of the CuO onto the NiO, proving excellent for better electrode and electrolyte conductivity at the interface. In addition, the surface area at both nano and macro scales was excellent, proving best in internal resistance. P8, despite having the highest internal resistance, managed to have lower electrode resistance.

This is likely due to the uniform porous structure of the electrode. P12 and P20 demonstrated the highest internal resistance. This is obvious since, as mentioned earlier, in the P12 sample, the welded CuO tape had a satisfactory connection to the oxidized NiO substrate, hence the higher internal resistance. However, the surface area for this sample proved excellent, which assisted in the formation of the EDL, as seen by the higher slope in mid-frequency, indicating the presence of EDL. P20, on the other hand, also demonstrated high internal resistance majority due to poor surface area on the macroscale due to increased particle agglomeration and fiber evaporation at high temperatures from larger laser pulse energy. Overall, P12 demonstrated excellent capacitive characteristics and was further analyzed by simulating with the equivalent open circuit as shown in Fig. 7c. The fit was made as shown in Fig. 7c, and various internal resistance was found as shown in Table 4.

**Table 4:** Simulation Results from curve fitting from EC-Lab Z-fit software for P12.

Components	Simulation results	
R_s_	7.417 Ω	
Q1	2.626 × 10^–3^ F*s^(a1–1)^	a1 = 0.646 7
R_ct_	1.471 Ω	
S2	10.3 Ω s^−1/2^	
Q2	5.887 × 10^–3^ F*s^(a2–1)^	a2 = 0.9187

The bode plot was analyzed to understand the phase angles of the components of the designed equivalent circuit with varying frequencies seen in Fig. 7d. From the phase angle analysis, it is observed that the constant phase of 45° at lower frequencies of P8 was dominant, proving the findings from the Nyquist analysis. This also demonstrates the Warburg impedance at the lower frequencies for P8. P15 and P20 at lower frequencies demonstrate stability at a phase angle around the high 60 s, progressing toward zero phase angle with a positive slope at mid frequencies, demonstrating the ion diffusion process. P12 was most promising to exhibit capacitive behavior at − 78°, which is very close to − 90° from the ideal capacitor, therefore, proving an excellent candidate for a pseudocapacitive device^71^. Figure 7e. Investigates the samples with impedance because of varying frequency. At lower frequencies, the device’s impedance is more significant; however, as the frequency has increased, the impedance has dropped significantly^72^. Of all the samples, P12 demonstrates the most negligible impedance compared to other samples.

To further study the conductivity of the electrode samples, four-point probe was utilized. The measurement of resistivity is shown in Table 5. The resistivity of the hybrid structure is more than that of pure Ni and Cu. The conductivity is measured by taking the reciprocal of the resistivity data acquired.

**Table 5:** Resistivity and conductivity of all samples.

Sample	Resistivity, $\rho$ (Ωm)	Conductivity, σ (S/m)
P8	$9.3077\times {10}^{-8}$	$1.0744\times {10}^{7}$
P12	$9.7857\times {10}^{-8}$	$1.0219\times {10}^{7}$
P15	$1.2842\times {10}^{-8}$	$7.7871\times {10}^{6}$
P20	$9.9803\times {10}^{-8}$	$1.0020\times {10}^{7}$

### Cycle retention, efficiency, and capacity

**Figure 8:**
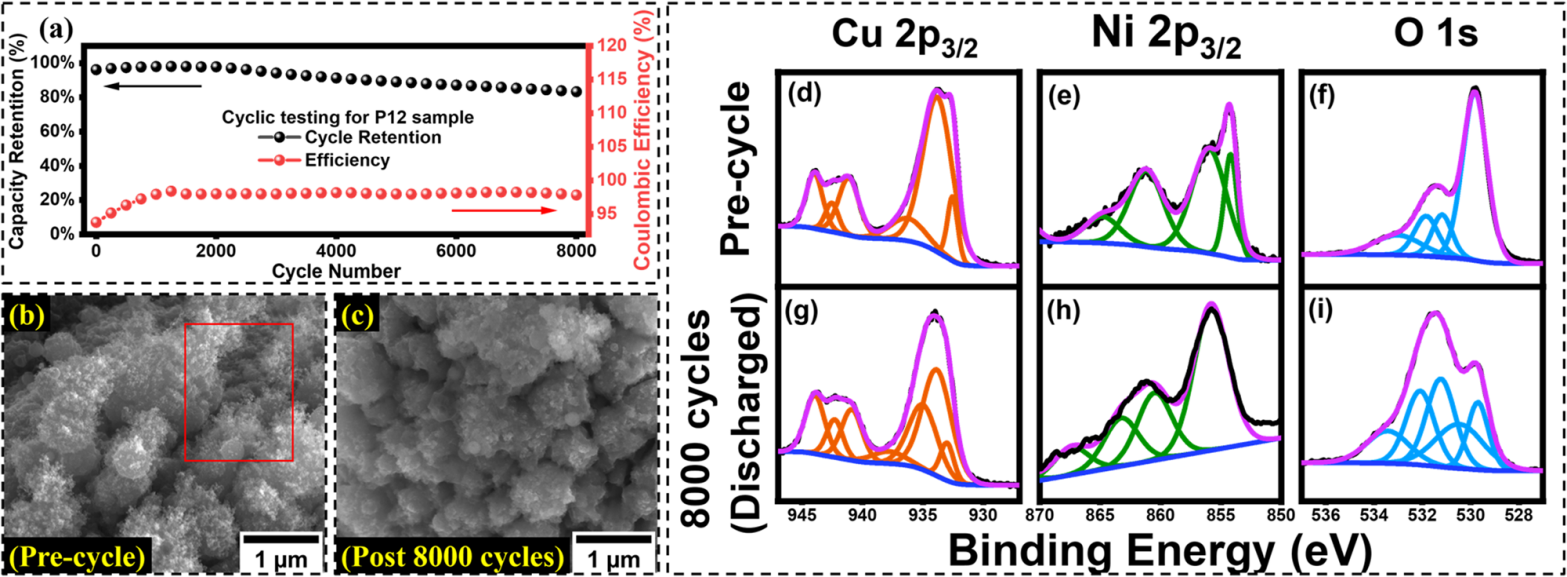
(**a**) Cycle retention of 83% achievable after 8000 cycles and 98% coulombic efficiency of the P12 sample. (**b**) SEM images of pre-cycle P12 sample (**c**) SEM images of P12 post 8000 cycle CD testing (**d**–**f**) Pre-cycle XPS deconvoluted signals (**g**–**i**) Post 8000 cycle XPS deconvoluted signals at a discharged state.

Capacity retention and columbic efficiency were analyzed with high current density charge–discharge of the P12 sample for 8000 cycles since it demonstrated the best electrical properties. A current density of 40 mA cm^−2^ was applied to the electrode resulting in rapid charge and discharge. The capacity retention of P12 was roughly 83% after 8000 cycles, which proved to be promising, as shown in Fig. 8a. The coulombic efficiency also proved excellent, maintaining a 97–98% efficiency during charge and discharge. The loss in capacity retention was investigated by conducting SEM on the pre-cycle and post-8000 cycle of the P12 sample surface morphology. Comparing Fig. 8b and c, it was easy to conclude that the drop in performance was due to a loss in nanofibers which reduce the specific surface area of the sample. Another reason for the drop in performance would be due to the battery material transition metals, which witness permanent phase over time transition and growing impedance due to poor rate capability. However, considering a less than 10-min fabrication, the cost of the electrodes could be extremely affordable and mass scalable due to rapid manufacturing. XPS was conducted on the samples after 8000 cycles of charge discharge. Figure 8d–f demonstrates Cu 2p_3/2_, Ni 2p_3/2,_ and O 1 s core levels before cycling. Here all the peaks are similar to as described in the structural characterization section of this manuscript. However, Fig. 7g–i demonstrates the deconvolution of the signals after 8000 cycles at discharge states. Both Cu and Ni show an increase in the ratio of Cu(II) Hydroxide at 934.75 and NiOOH at ~ 855 after the reduction reaction due to discharging state. The O 1 s peak demonstrates a drop in lattice oxygen and increases in the O–H bonding group.

**Figure 9:**
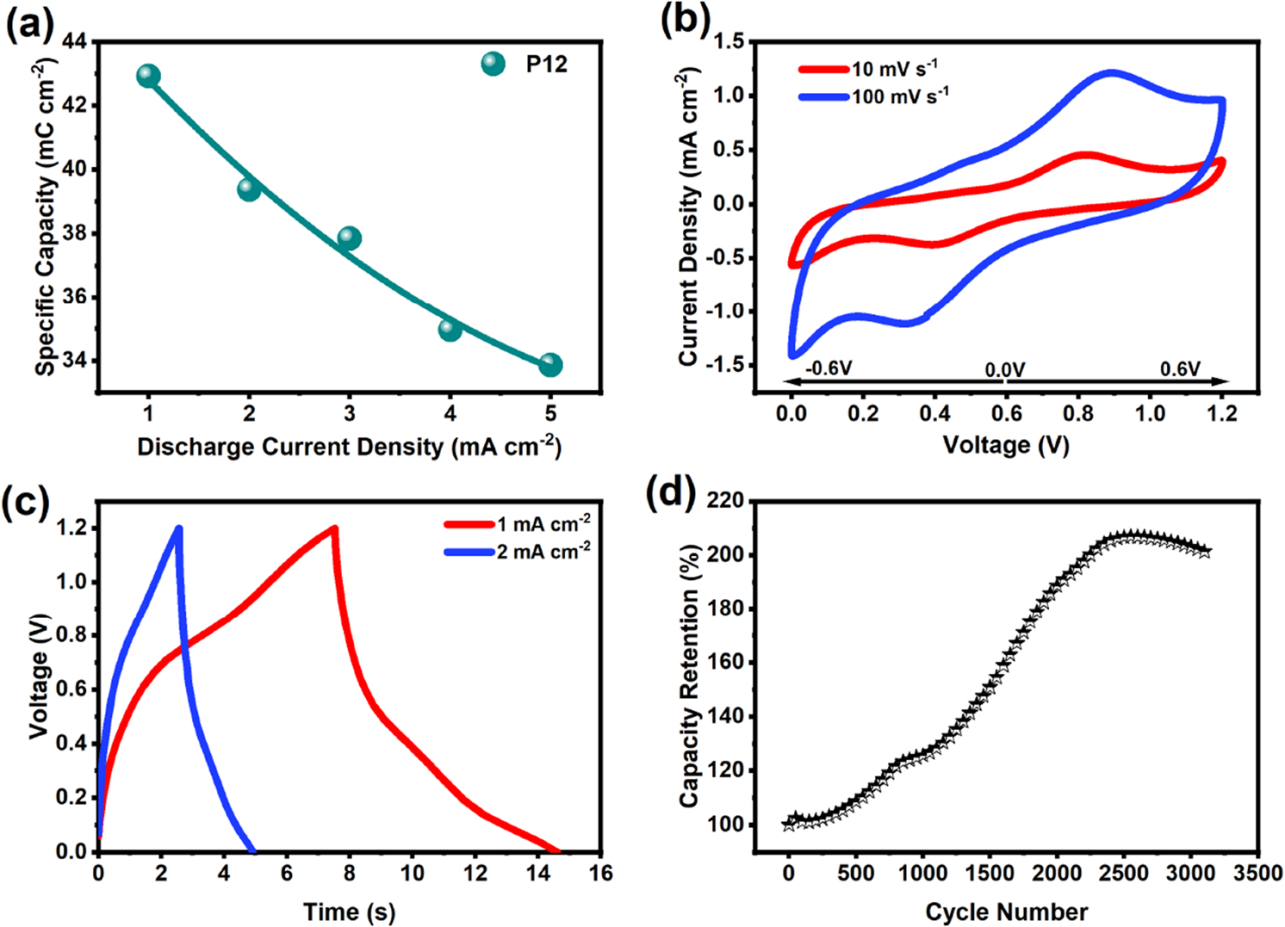
(**a**) The areal capacitance of P12 is calculated with CV at different scan speeds. (**b**) The CV almost demonstrates excellent reversibility at both 10 and 100 mV s^−1^ scan rate (**c**) The charge–discharge curve is almost non-linear proving some pseudocapacitive characteristics (**d**) Interesting observations were made with the capacity almost doubling after the 2500 cycle, after which degradation is evident.

The areal capacitance was calculated using discharge current densities. A specific capacitance of 25.8 mC cm^−2^ was achievable at a current density of 1 mA cm^−2^, proving excellent performance. However, the specific capacity decreases as the scan rate is increased as seen in Fig. 9a. This is evident from many works of literature published, as this is most commonly due to the insufficient time for ions to diffuse into the electrode surface. A coin cell setup was proposed with a symmetric assembly of the P12 electrodes with a separator soaked in a high concentration 2 M KOH electrolyte. A series of CV, GCD, and capacity tests were conducted.

Presented in Fig. 9b is the CV for the P12 coin cell at a scan speed of 10 mV s^−1^ and 100 mV s^−1^. The suitable potential window was chosen to be 1.2 V, demonstrating maximum stability, dominant redox peaks, and perfect reversibility. The expanded voltage provided symmetric anodic and cathodic peaks, which were desirable for symmetric cells. GCD tests were also conducted on the coin cells as shown in Fig. 9c. The areal capacitance was found to be 16 mF cm^−2^ at a 2 mA cm^−2^ scan rate exhibiting excellent charge storage characteristics. The discharge capacity of the coin cell was monitored by the cycling of GCD at 5 mA cm^−2^ for 3000 cycles seen in Fig. 9d. Interesting results were obtained, demonstrating an increase in the discharge capacity of the symmetric cell of P12. The capacity was almost doubled until 2500 cycles, after which the capacity decreased. Many works of literature that made use of laser for the formation of electrodes, such as laser deposition, have reported an increase in retention due to the activation of the active material over long cycling, which could be due to the surface area modification over time^73–75^. These retention results are significantly different as compared to the CD test using the three-electrode setup. However, the two-electrode setup is based on the most realistic model of the design.

The as-synthesized sample, which was irradiated with 12W of laser energy, exhibited the formation of a hybrid NiO/CuO structure. The hybrid structure displayed synergistic effects and improved performance in the electrochemical behavior, as compared to single-phase NiO or CuO. This is a remarkable achievement, as it was achieved within a 2-step process that took less than 10 min for a 1 cm^2^ active oxide surface. This fabrication process is extremely cost-effective, as well as a chemical-free, green synthesis approach. In the current state of the art of supercapacitor technology, many research works demonstrate remarkable electrochemical results of the supercapacitor; however, the fabrication practices are not practical, as some research reports a fabrication time of 24 + hours with a multi-step process. The novelty of this work lies in its simplicity of combining two earth-abundant transition metals to form a hybrid structure with the help of a pulsed laser, which has not yet been reported in the literature.

## Conclusion

In this work, the ULPING method was presented to develop a hybrid structure for hybrid structure. Pulse ionization fusion of high purity Cu tape onto Ni sheet to form a CuO/NiO hybrid structure for electrode was possible via picosecond laser. Mainly analysis of morphology with a variation of laser intensity or pulse energy was demonstrated. This method created self-standing, binder-free 3D nanostructures which exhibit remarkable performance as a candidate for the pseudocapacitive electrode. At 12W average power, the best electrode characteristics were observed, such as excellent synergistic effects, great surface area in both micro and nanoscale, uniform surface area distribution, high porosity, superior conductivity, and excellent reversibility of the reactions. An areal capacitance of 25.8 mC cm^−2^ was achievable at a current density of 1 mA cm^−2^ proving to be excellent considering the fabrication took less than 10 min following a simple 2-step process. The capacity retention of 83% was achievable after 8000 cycles of charge–discharge. The good retention well compensates for the affordable cell that could be manufactured at an economical and scalable method using the approach used in this work. This work promotes the idea of green synthesis and scalable fabrication of electrodes with the ULPING method.

## Data Availability

The datasets used and/or analyzed during the current study are included in the published article. The raw data used are available upon request from the corresponding author.

## References

[1] Olabi A (2017). Renewable Energy and Energy Storage Systems.

[2] Foley A, Olabi AG (2017). Renewable Energy Technology Developments, Trends and Policy Implications that can Underpin the Drive for Global Climate Change.

[3] Gielen D, Boshell F, Saygin D, Bazilian MD, Wagner N, Gorini R (2019). The role of renewable energy in the global energy transformation. Energ. Strat. Rev..

[4] Aziz SB, Hamsan MH, Kadir MF, Karim WO, Abdullah RM (2019). Development of polymer blend electrolyte membranes based on chitosan: Dextran with high ion transport properties for EDLC application. Int. J. Mol. Sci..

[5] Asnawi AS (2020). Glycerolized Li^+^ ion conducting chitosan-based polymer electrolyte for energy storage EDLC device applications with relatively high energy density. Polymers.

[6] Conway B, Pell W (2003). Double-layer and pseudocapacitance types of electrochemical capacitors and their applications to the development of hybrid devices. J. Solid State Electrochem..

[7] Lin X, Khosravinia K, Hu X, Li J, Lu W (2021). Lithium plating mechanism, detection, and mitigation in lithium-ion batteries. Prog. Energy Combust. Sci..

[8] Sajjad M (2021). Recent trends in transition metal diselenides (XSe_2_: X= Ni, Mn, Co) and their composites for high energy faradic supercapacitors. J. Energy Storage.

[9] Sajjad M, Lu W (2021). Covalent organic frameworks based nanomaterials: Design, synthesis, and current status for supercapacitor applications: A review. J. Energy Storage.

[10] Sajjad M, Cheng F, Lu W (2021). Research progress in transition metal chalcogenide based anodes for K-ion hybrid capacitor applications: A mini-review. RSC Adv..

[11] Sundriyal S, Kaur H, Bhardwaj SK, Mishra S, Kim K-H, Deep A (2018). Metal-organic frameworks and their composites as efficient electrodes for supercapacitor applications. Coord. Chem. Rev..

[12] Salunkhe RR, Kaneti YV, Yamauchi Y (2017). Metal–organic framework-derived nanoporous metal oxides toward supercapacitor applications: Progress and prospects. ACS Nano.

[13] Jayalakshmi M, Balasubramanian K (2008). Simple capacitors to supercapacitors-an overview. Int. J. Electrochem. Sci.

[14] Chen R, Yu M, Sahu RP, Puri IK, Zhitomirsky I (2020). The development of pseudocapacitor electrodes and devices with high active mass loading. Adv. Energy Mater..

[15] Bu IY, Huang R (2017). Fabrication of CuO-decorated reduced graphene oxide nanosheets for supercapacitor applications. Ceram. Int..

[16] Wang Y, Guo J, Wang T, Shao J, Wang D, Yang Y-W (2015). Mesoporous transition metal oxides for supercapacitors. Nanomaterials.

[17] Liu C, Li F, Ma LP, Cheng HM (2010). Advanced materials for energy storage. Adv. Mater..

[18] Sajjad M, Khan MI, Cheng F, Lu W (2021). A review on selection criteria of aqueous electrolytes performance evaluation for advanced asymmetric supercapacitors. J. Energy Storage.

[19] Jung H-G, Yoon CS, Prakash J, Sun Y-K (2009). Mesoporous anatase TiO_2_ with high surface area and controllable pore size by F−-ion doping: Applications for high-power Li-ion battery anode. J. Phys. Chem. C.

[20] Baumann AE, Burns DA, Liu B, Thoi VS (2019). Metal-organic framework functionalization and design strategies for advanced electrochemical energy storage devices. Commun. Chem..

[21] Ma Y (2021). Recent advances in transition metal oxides with different dimensions as electrodes for high-performance supercapacitors. Adv. Compos. Hybrid Mater..

[22] Low WH, Khiew PS, Lim SS, Siong CW, Ezeigwe ER (2019). Recent development of mixed transition metal oxide and graphene/mixed transition metal oxide based hybrid nanostructures for advanced supercapacitors. J. Alloys Compd..

[23] Sajjad M (2022). A new CuSe-TiO_2_-GO ternary nanocomposite: Realizing a high capacitance and voltage for an advanced hybrid supercapacitor. Nanomaterials.

[24] Sajjad M (2023). CdO nanocubes decorated on rGO sheets as novel high conductivity positive electrode material for hybrid supercapacitor. J. Alloys Compd..

[25] Pazhamalai P, Krishnamoorthy K, Manoharan S, Mariappan VK, Kim S-J (2022). Monolithic integration of MoS_2_ quantum sheets on solid electrolyte for self-charging supercapacitor power cell governed by piezo-ionic effect. Sustain. Mater. Technol..

[26] Mishra D, Kim S, Kumar N, Krishnaiah M, Jin SH (2023). Self-discharge mitigated supercapacitors via hybrid CuO-nickel sulfide heterostructure for energy efficient, wireless data storage application. J. Mater. Sci. Technol..

[27] Khot M, Kiani A (2022). A review on the advances in electrochemical capacitive charge storage in transition metal oxide electrodes for pseudocapacitors. Int. J. Energy Res..

[28] Li B, Zheng M, Xue H, Pang H (2016). High performance electrochemical capacitor materials focusing on nickel based materials. Inorg. Chem. Front..

[29] Li R, Lin Z, Ba X, Li Y, Ding R, Liu J (2016). Integrated copper–nickel oxide mesoporous nanowire arrays for high energy density aqueous asymmetric supercapacitors. Nanoscale Horiz..

[30] Fang Z, ur Rehman S, Sun M, Yuan Y, Jin S, Bi H (2018). Hybrid NiO–CuO mesoporous nanowire array with abundant oxygen vacancies and a hollow structure as a high-performance asymmetric supercapacitor. J. Mater. Chem. A.

[31] Chakraborty I, Chakrabarty N, Senapati A, Chakraborty AK (2018). CuO@ NiO/Polyaniline/MWCNT nanocomposite as high-performance electrode for supercapacitor. J. Phys. Chem. C.

[32] Huang M, Li F, Zhang YX, Li B, Gao X (2014). Hierarchical NiO nanoflake coated CuO flower core–shell nanostructures for supercapacitor. Ceram. Int..

[33] Jamwal NS, Kiani A (2022). Gallium oxide nanostructures: A review of synthesis, properties and applications. Nanomaterials.

[34] Gholami A, Kiani A (2020). Laser-induced nanofibrous titania film electrode: A new approach for energy storage materials. J. Energy Storage.

[35] Gholami A, Yim C-H, Kiani A (2021). Electrochemical performance of Titania 3D nanonetwork electrodes induced by pulse ionization at varied pulse repetitions. Nanomaterials.

[36] Khot M, Kiani A (2022). Formation of NiO thin-film via picosecond laser pulses for energy storage electrode fabrication. Optical Interference Coatings.

[37] Khot M, Kiani A (2022). Synthesis of self-grown nanostructured NiO via pulse ionization for binderless psuedocapacitor electrode. J. Energy Storage.

[38] Khosravinia K, Kiani A (2013). Unlocking pseudocapacitors prolonged electrode fabrication via ultra-short laser pulses and machine learning. iScience.

[39] Palneedi H (2018). Laser irradiation of metal oxide films and nanostructures: Applications and advances. Adv. Mater..

[40] Kiani A, Venkatakrishnan K, Tan B (2009). Micro/nano scale amorphization of silicon by femtosecond laser irradiation. Opt. Express.

[41] Kiani A, Venkatakrishnan K, Tan B (2010). Direct laser writing of amorphous silicon on Si-substrate induced by high repetition femtosecond pulses. J. Appl. Phys..

[42] Paladiya C, Kiani A (2018). Nano structured sensing surface: Significance in sensor fabrication. Sens. Actuators B Chem..

[43] Abed MM, Gaspari F, Kiani A (2020). Optical properties of Si/SiO_2_ nano structured films induced by laser plasma ionization deposition. Opt. Commun..

[44] Joshi S, Kiani A (2021). Hybrid artificial neural networks and analytical model for prediction of optical constants and bandgap energy of 3D nanonetwork silicon structures. Opto Electron. Adv..

[45] AlMarzooqi FA, Bilad M, Mansoor B, Arafat HA (2016). A comparative study of image analysis and porometry techniques for characterization of porous membranes. J. Mater. Sci..

[46] Kakani V (2020). Facile synthesis of CuO/NiO/nitrogen doped rGO by ultrasonication for high performance supercapacitors. J. Alloys Compd..

[47] Dey S, Santra S, Midya A, Guha PK, Ray SK (2017). Synthesis of Cu_x_ Ni_(1–x)_O coral-like nanostructures and their application in the design of a reusable toxic heavy metal ion sensor based on an adsorption-mediated electrochemical technique. Environ. Sci. Nano.

[48] Srivastava N, Srivastava P (2010). Realizing NiO nanocrystals from a simple chemical method. Bull. Mater. Sci..

[49] Meghana S, Kabra P, Chakraborty S, Padmavathy N (2015). Understanding the pathway of antibacterial activity of copper oxide nanoparticles. RSC Adv..

[50] Panzeri G, Cristina M, Jagadeesh M, Bussetti G, Magagnin L (2020). Modification of large area Cu_2_O/CuO photocathode with CuS non-noble catalyst for improved photocurrent and stability. Sci. Rep..

[51] Sajjad M (2022). A novel high-performance all-solid-state asymmetric supercapacitor based on CuSe nanoflakes wrapped on vertically aligned TiO_2_ nanoplates nanocomposite synthesized via a wet-chemical method. J. Energy Storage.

[52] Sajjad M (2021). Fabrication of 1.6 V hybrid supercapacitor developed using MnSe_2_/rGO positive electrode and phosphine based covalent organic frameworks as a negative electrode enables superb stability up to 28,000 cycles. J. Energy Storage.

[53] Sajjad M, Lu W (2021). Regulating high specific capacitance NCS/α-MnO_2_ cathode and a wide potential window α-Fe_2_O_3_/rGO anode for the construction of 2.7 V for high performance aqueous asymmetric supercapacitors. J. Energy Storage.

[54] Huang W (2017). 3D NiO hollow sphere/reduced graphene oxide composite for high-performance glucose biosensor. Sci. Rep..

[55] Wang H-Q (2017). In situ growth of NiO nanoparticles on carbon paper as a cathode for rechargeable Li–O_2_ batteries. RSC Adv..

[56] Salunkhe P, AV MA, Kekuda D (2020). Investigation on tailoring physical properties of Nickel Oxide thin films grown by dc magnetron sputtering. Mater. Res. Express.

[57] Lv W, Li L, Meng Q, Zhang X (2020). Molybdenum-doped CuO nanosheets on Ni foams with extraordinary specific capacitance for advanced hybrid supercapacitors. J. Mater. Sci..

[58] Yuan J, Zhang J-J, Yang M-P, Meng W-J, Wang H, Lu J-X (2018). CuO nanoparticles supported on TiO_2_ with high efficiency for CO_2_ electrochemical reduction to ethanol. Catalysts.

[59] Sapkota KP, Lee I, Hanif MA, Islam MA, Akter J, Hahn JR (2020). Enhanced visible-light photocatalysis of nanocomposites of copper oxide and single-walled carbon nanotubes for the degradation of methylene blue. Catalysts.

[60] Pazhamalai P, Krishnamoorthy K, Mariappan VK, Kim S-J (2019). Blue TiO_2_ nanosheets as a high-performance electrode material for supercapacitors. J. Colloid Interface Sci..

[61] Wang J, Polleux J, Lim J, Dunn B (2007). Pseudocapacitive contributions to electrochemical energy storage in TiO_2_ (anatase) nanoparticles. J. Phys. Chem. C.

[62] Augustyn V, Simon P, Dunn B (2014). Pseudocapacitive oxide materials for high-rate electrochemical energy storage. Energy Environ. Sci..

[63] Brezesinski T, Wang J, Tolbert SH, Dunn B (2010). Ordered mesoporous α-MoO3 with iso-oriented nanocrystalline walls for thin-film pseudocapacitors. Nat. Mater..

[64] Nk MS, Alex C, Jana R, Datta A, John NS (2022). Remarkable CO_x_ tolerance of Ni^3+^ active species in a Ni_2_O_3_ catalyst for sustained electrochemical urea oxidation. J. Mater. Chem. A.

[65] Babar NUA, Joya KS (2020). Spray-coated thin-film Ni-oxide nanoflakes as single electrocatalysts for oxygen evolution and hydrogen generation from water splitting. ACS Omega.

[66] Shi W (2020). Enabling superior sodium capture for efficient water desalination by a tubular polyaniline decorated with Prussian blue nanocrystals. Adv. Mater..

[67] Dahotre NB, Harimkar S (2008). Laser Fabrication and Machining of Materials.

[68] Chatterjee S, Ray A, Mandal M, Das S, Bhattacharya SK (2020). Synthesis and characterization of CuO–NiO nanocomposites for electrochemical supercapacitors. J. Mater. Eng. Perform..

[69] Zhang YX, Kuang M, Wang JJ (2014). Mesoporous CuO–NiO micropolyhedrons: facile synthesis, morphological evolution and pseudocapcitive performance. CrystEngComm.

[70] Hosseini SG, Abazari R (2015). A facile one-step route for production of CuO, NiO, and CuO–NiO nanoparticles and comparison of their catalytic activity for ammonium perchlorate decomposition. RSC Adv..

[71] Tamilarasan P, Ramaprabhu S (2014). Ionic liquid-functionalized partially exfoliated multiwalled carbon nanotubes for high-performance supercapacitors. J. Mater. Chem. A.

[72] Xu R, Guo F, Cui X, Zhang L, Wang K, Wei J (2015). High performance carbon nanotube based fiber-shaped supercapacitors using redox additives of polypyrrole and hydroquinone. J. Mater. Chem. A.

[73] Lacerda JN, Franceschini D, Ponzio E, Esteves LM, Guimarães RB, Xing Y (2020). Manganese oxide nanofoam prepared by pulsed laser deposition for high performance supercapacitor electrodes. Mater. Chem. Phys..

[74] Guerra A (2019). ZnO/Carbon nanowalls shell/core nanostructures as electrodes for supercapacitors. Appl. Surf. Sci..

[75] Nikam S (2020). Pulsed laser deposited CoFe_2_O_4_ thin films as supercapacitor electrodes. RSC Adv..

